# Families with high-risk characteristics and diagnoses of attention-deficit/hyperactivity disorder, autism spectrum disorder, intellectual disability, and learning disability in children: A national birth cohort study

**DOI:** 10.3389/fpsyg.2022.758032

**Published:** 2022-10-06

**Authors:** For-Wey Lung, Po-Fei Chen, Li-Jong Shen, Bih-Ching Shu

**Affiliations:** ^1^Calo Psychiatric Center, Pingtung County, Taiwan; ^2^National Defense Medical Center, Graduate Institute of Medical Science, Taipei, Taiwan; ^3^International Graduate Program of Education and Human Development, National Sun Yat-sen University, Kaohsiung, Taiwan; ^4^Institute of Education, National Sun Yat-sen University, Kaohsiung, Taiwan; ^5^Department of Mental and Oral Health, Ministry of Health and Welfare, Taipei, Taiwan; ^6^Institute of Allied Health Sciences, Department of Nursing, College of Medicine, National Cheng Kung University, Tainan, Taiwan

**Keywords:** high-risk family, Taiwan Birth Cohort Study (TBCS), attention-deficit/hyperactivity disorder (ADHD), intellectual disability (ID), learning disorder (LD)

## Abstract

**Background:**

A national birth cohort study was used to investigate whether high-risk family factors at 1.5-year-olds can increase the risk of attention-deficit/hyperactivity disorder (ADHD) diagnosis when children reach 5.5 years. The pathway relationship of high-risk family factors, children's developmental conditions, risk of autism spectrum disorder (ASD), and diagnosis of intellectual disability (ID), learning disability (LD), and ASD was also investigated.

**Methods:**

The 1.5-, 3- and 5.5-year-old Taiwan Birth Cohort Study (TBCS) dataset was used (N = 19,185). The high-risk familial factor was measured using five questions assessing whether parents are currently unmarried, unemployed, do not have any social insurance, perceive a “very heavy” economic childcare burden, and at least one of the parents has a disability certification. Developmental conditions were assessed using the Taiwan Birth Cohort Study—Developmental Instrument (TBCS-DI), and ASD risk was measured using the Modified Checklist of Autism in Toddlers. Data on ADHD, ID, LD, and ASD diagnoses were collected at age 5.5. The odds ratio model investigated whether children from families with high-risk factors at 1.5-years were at increased risk of ADHD, ID, LD, or ASD diagnosis at 5.5-years, compared to those children from families without such risks. Structural equation modeling investigated the logistic regression pathway relationship of high-risk familial characteristics, children's developmental conditions, autism screening, and diagnosis.

**Results:**

In the national birth cohort dataset of 19,185 children, 2070 (10.8%) met at least one of the high-risk familial factors. Children who met one high-risk familial factor had a 1.21-fold increased risk for ADHD diagnosis, 1.36-fold increased risk for LD diagnosis, and 1.80-fold increased risk for ASD diagnosis, compared to children from families without risks. High-risk familial factors directly increased the risk of ADHD and ID diagnosis, and indirectly increased the risk of ADHD, ID, LD, and ASD diagnosis through the mediating factor of children's development.

**Conclusions:**

Children who met more high-risk familial characteristics were at higher risk of ADHD, ID, LD, and ASD diagnosis. Development at three years was predictive of diagnosis at 5.5 years. Thus, developmental screening at age three is vital for interventions. Preventive, family-focused, and/or child-rearing services for at-risk families are important for improving outcomes for these children and their families.

## Introduction

Parent and family function have a significant influence on the developmental conditions of children, and dysfunction is strongly associated with psychopathy in children (Ingoldsby et al., 2001; D'Onofrio and Emery, 2019). An increasing number of children experience family instability (parental divorce or separation) and this has become a major public health problem (D'Onofrio and Emery, 2019). Although parent and family function are strongly associated with psychopathology in children, children's psychiatric diagnosis may also in turn increase the stress on family members and impair their function (Wallander et al., 2006). Additionally, family dysfunction, such as marital instability, is not a single risk factor but may imply economic struggles, less effective parenting, and interparental conflict (Emery, 2016).

Among psychiatric diagnoses, parental distress and family dysfunction have been found to predict the development of children with intellectual disabilities (ID) (Wallander et al., 2006). Although inattention and hyperactivity in attention-deficit/hyperactivity disorder (ADHD) are highly heritable (Nikolas and Burt, 2010), gene-environment interaction has also been shown to have an adverse effect on the family environment and to have a significant influence in children with ADHD and associated psychiatric, cognitive, and psychosocial impairments (Biederman et al., 2004). Similarly, a study of adoption showed that better family cohesion and adaptability mediate the influence of family environment on children's ADHD symptoms (Crea et al., 2014). Parents of children with autism spectrum disorder (ASD) have reported less dyadic consensus and perceived less marital satisfaction, family adaptability, and cohesion than parents of children with more typical development (Gau et al., 2012). Studies of learning disorders (LD) have also found harmonious familial relationships, and available guidance and resources can assist with childcare and alleviate parental stress (Karande et al., 2007; Buschgens et al., 2008; Chien and Lee, 2013).

Genes can have an interactive effect on the environment. However, most previous studies on the association of family function and children's psychiatric diagnosis were of cross-sectional design, thus unable to distinguish whether parent and family function is the cause or consequence of psychopathology. The Taiwan Birth Cohort Study (TBCS), therefore, aimed to record and assess the health trajectory of children born in the twenty-first century and investigate the influence of the social environment on child health. Thus, the TBCS dataset was used to investigate whether high-risk family characteristics at age 1.5 years increased the risk of ADHD diagnosis when the children reached 5.5 years. Furthermore, the pathway relationship of high-risk family factors, children's developmental conditions, risk of ASD, diagnosis of ID, LD, and ASD was also investigated.

## Materials and methods

### Participants

The Taiwan Birth Cohort Study aimed to develop a nationally representative cohort database, with all children born in 2005 eligible to participate, with no exclusion criteria, using a national household probability and a two-stage stratified random sampling method. The township was used as the primary sampling unit; 369 were stratified into 12 strata according to the degree of urbanization and fertility rate, and 89 were selected using systematic random sampling (Chang et al., 2021). The probability proportional to size sampling method was used to draw newborns from the sampled townships at the first stage (Chang et al., 2021). In the final sample, 21,248 children (selection rate of 11.7%) were selected when the children were 6 months old (Chang et al., 2021). At 1.5 years of age, 20,172 (94.94%) families agreed to remain in the study; at 3 years of age, 19,910 (94%) continued with the study, and 19,721 (92.81%) families agreed to participate in the study when their children were aged 5.5.

The protocol of this study was approved by the Institutional Review Board of a teaching hospital in Taiwan and was in accordance with the Declaration of Helsinki. Written informed consent was obtained from the parent or main caregiver of the participating children at each stage of the study.

### Materials

A trained researcher visited the homes of the participating families at each stage of the TBCS study. The researchers asked the parents questions from the structural TBCS interview booklet, which included questions designed to collect data on variables that may affect children's health and development (Chang et al., 2021). Factors collected included the child's sex, high-risk family characteristics, developmental condition of the children, M-CHAT screening, and whether the child had ever received ADHD, ID, LD, or ASD diagnosis. Sex was controlled as a confounding factor in the SEM analysis. Demographic information about the parents and children was collected when the children were 6 months old. High-risk family information was only collected at age 1.5. The developmental status of the children (The Taiwan Birth Cohort Study–Developmental Instrument; TBCS-DI) was obtained at ages 1.5, 3, and 5.5. In addition, when the children were 5.5 years old, the Modified Checklist of Autism in Toddlers (M-CHAT) was assessed. Information on whether the child had ever been diagnosed with ADHD, ID, LD, or ASD was also collected when they were 5.5 years old. All the above questionnaires were included in the TBCS interview booklet, and the data was compiled after the 5.5 year data was collected.

#### High-risk family

High-risk families were assessed using the following questions: “Parents are currently unmarried (did not marry, divorced, or deceased)”; “Parents are currently unemployed”; “Parents do not have any social insurance”; “Parents perceive a ‘very heavy' economic childcare burden”; and “At least one of the parents has a disability certification”. “Yes” was coded as “1” and “No” as “0”. Scores were added together to form the high-risk factor. With the amendment of the Child and Youth Welfare Act in 2003 in Taiwan, the responsibility of the government to protect children aged 0–17 and promote their wellbeing was enhanced, including providing emergency placement of children who are unable to avoid immediate danger concerning their lives, bodies, or freedom from their primary caregivers. This “high-risk family evaluation” scale was developed by experts in psychiatry, psychology, nursing, public health, education, and psychometric statistics for screening children who may need a referral to Child Welfare Services to prevent child maltreatment and enforced along with the High-risk Family Service Project in 2005 (Shih and Song, 2006).

#### Developmental measurement

The TBCS-DI is a short, culturally sensitive, parental-report developmental instrument used to measure children's developmental conditions at ages 1.5, 3, and 5.5. The TBCS-DI includes 17 items on the 1.5-year scale, 19 on the 3-year scale, and 16 on the 5.5-year scale. The parental response was obtained on a 3-point Likert scale. The 1.5-, 3-, and 5.5-year scales all showed good construct, predictive, and content validity (Lung et al., 2008, 2010, 2011), with higher scores implying better development.

#### Autism screening

M-CHAT is a parent-reported 23-item screening instrument designed to screen children at high risk of ASD in the general population (Robins et al., 2001).

The M-CHAT was originally developed for screening children aged between 16 and 30 months. However, since the prevalence rate of ASD is highest in the age group of 6 to 11-year-olds in Taiwan, which is later than that of the US (Lai et al., 2012), the application of the M-CHAT in children aged 5.5 was investigated in a community study in Taiwan and found that children who failed any 10 of the 23 items were considered to be at high risk of ASD (Lung et al., 2017). Therefore, a cutoff of 13/14 items was used for the present study.

### Statistical analysis

Bayesian analysis was used to replace missing data. It accounts for multiple sources of correlation and is a multiple imputation method based on item response theory. The odds ratio was used to investigate whether children from families with high-risk familial factors at 1.5-years of age were at increased risk of ADHD, ID, LD, or ASD diagnosis at 5.5 years, compared to those children from families without risk factors. The demographic distribution of the children's odds ratio of increased risk of diagnosis in high-risk families was analyzed using Statistical Package for the Social Sciences (SPSS) 20.0 for Windows (SPSS Inc., Chicago, IL, USA).

The pathway relationship of the comparison of children from high-risk familial characteristics with those without such risks, children's developmental conditions, and autism screening and diagnosis were analyzed using a structural equation model (SEM). The Analysis of Moment Structures 7.0 statistical software package (SPSS) was used to process the results of the SEM analysis. The SEM models had p > 0.5, adjusted goodness-of-fit index (AGFI) > 0.9, and root mean square error of approximation (RMSEA) <0.08, which implied that the null model approximated the real structure. Only the parsimonious model is presented, meaning that only statistically significant (p < 0.05) variables and pathways were presented, for easier reading and interpretation.

## Results

The demographic distribution of the children and parents is shown in Table 1. Of the 19,185 children in our study, 2070 (10.8%) met at least one of the high-risk familial characteristics. Of the total, 177 (0.9%) reported being diagnosed with ADHD, 508 (2.6%) with ID, 283 (1.5%) LD, and 86 (0.4%) with ASD.

**Table 1:** Demographic distribution of children and parents (*N* = 19,185).

**Variable**	**n (%)**
Child sex	
Boy	10,085 (52.6)
Girl	9,100 (47.4)
High risk factors	2,070 (10.8)
Diagnosis at 5.5 yr	
Attention deficit/hyperactivity disorder	177 (0.9)
Intellectual disability	508 (2.6)
Learning disorder	283 (1.5)
Autism spectrum disorder	86 (0.4)
Maternal education:	
Elementary school	735 (3.9)
Middle school	1,984 (10.3)
High school	7,689 (40.1)
University/college	8,109 (42.3)
Graduate school	666 (3.5)
Paternal education:	
Elementary school	262 (1.4)
Middle school	2,313 (12.1)
High school	7,637 (39.8)
University/college	7,513 (39.2)
Graduate school	1,460 (7.6)
Variable (range)	Mean (SD)
Parental age at childbirth (yr)	
Mother's age (14–49)	29.41 (4.85)
Father's age (17–80)	33.26 (5.41)

Children who met any of the five high-risk familial characteristics (parents unmarried, unemployed, having no social insurance, perceiving a very heavy economic childcare burden, or having disability certification) were at increased risk of ADHD, ID, LD, and ASD (Table 2). Children who met at least one of the high-risk characteristics were at 1.21-fold increased risk for ADHD diagnosis (95% confidence interval [CI]: 1.53–3.18), 1.24-fold increased risk for ID diagnosis (95% CI: 1.80–2.79), 1.36-fold increased risk for LD diagnosis (95% CI: 1.77–3.14), 1.05-fold increased risk for ASD diagnosis (95% CI: 1.20–3.49), and 3.89-fold increased risk to be screened positive for ASD (95% CI: 1.77–3.14). Those who met two or more of the high-risk family characteristics were at 1.17-fold increased risk for ADHD diagnosis (95% CI: 0.95–4.93), 1.75-fold increased risk for ID diagnosis (95% CI: 1.75–4.33), 2.81-fold increased risk for LD diagnosis (95% CI: 2.27–6.39), 1.22-fold increased risk for ASD diagnosis (95% CI: 0.70–7.06), and 4.88-fold increased risk to screen positive for ASD (95% CI: 2.10–16.50). The relative risks of diagnosis in those who met more than three high-risk family characteristics, and the relative risk for each high-risk characteristic, are also shown in Table 2.

**Table 2:** High-risk family characteristics and relative risk of attention-deficit/hyperactivity disorder (ADHD), autism spectrum disorder (ASD), intellectual disability (ID), and learning disability (LD) diagnosis (*N* = 19,185).

		**ADHD**	**ID**	**LD**	**ASD**	**M-CHAT ≧14**
	**n (%)**	**OR (95% CI)**	**OR (95% CI)**	**OR (95% CI)**	**OR (95% CI)**	**OR (95% CI)**
No. of high-risk family characteristics met						
1	2,070 (10.8)	2.21 (1.53–3.18)	2.24 (1.80–2.79)	2.36 (1.77–3.14)	2.05 (1.20–3.49)	4.89 (1.77–3.14)
2	309 (1.6)	2.17 (0.95–4.93)	2.75 (1.75–4.33)	3.81 (2.27–6.39)	2.22 (0.70–7.06)	5.88 (2.10–16.50)
3	64 (0.3)	5.36 (1.66–17.23)	4.56 (2.07–10.06)	3.31 (1.03–10.61)	—	—
Parents unmarried	610 (3.2)	2.87 (1.68–4.89)	2.03 (1.40–2.95)	1.60 (0.93–2.75)	1.49 (0.54–4.07)	2.13 (0.66–6.89)
Parents unemployed	721 (3.8)	1.71 (0.92–3.16)	2.00 (1.42–2.84)	2.08 (1.33–3.27)	1.59 (0.64–3.92)	3.14 (1.24–7.96)
Parents have no social insurance	65 (0.3)	1.68 (0.23–12.19)	1.17 (0.29–4.79)	3.26 (1.02–10.43)	—	—
Parents perceive a very heavy economic childcare burden	670 (3.5)	2.21 (1.25–3.92)	3.03 (2.23–4.12)	3.25 (2.20–4.81)	2.08 (0.91–4.79)	6.80 (3.27–14.15)
Parent has disability certification	384 (2.0)	1.73 (0.76–3.93)	1.95 (1.22–3.12)	2.61 (1.51–4.51)	3.05 (1.23–7.57)	4.70 (1.68–13.18)

OR, Odds ratio; CI, Confidence interval.

**Figure 1:**
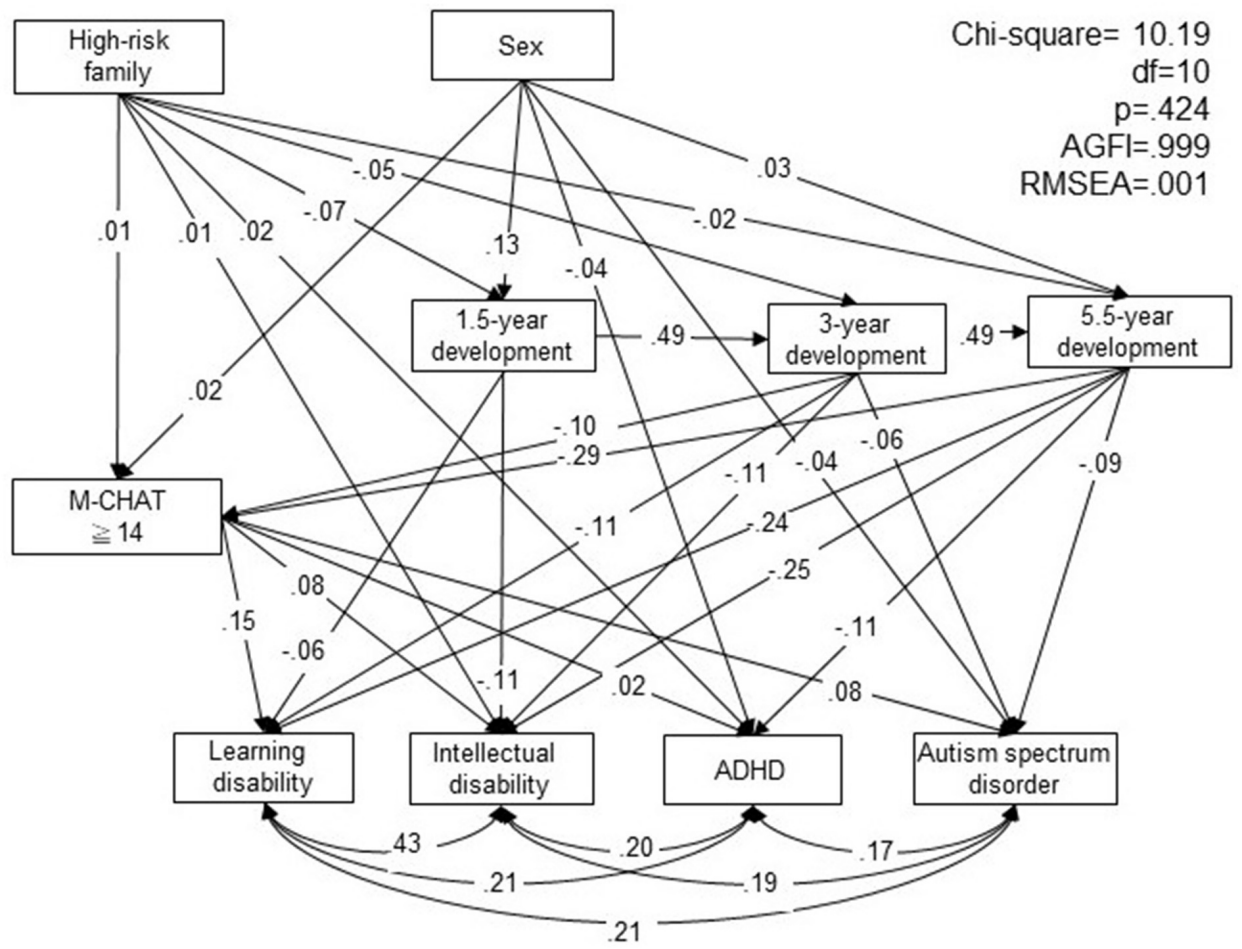
Pathway relationship of high-risk family characteristics, children's developmental conditions, autism spectrum disorder (ASD) screening, diagnosis of attention-deficit/hyperactivity Disorder (ADHD), intellectual disability, learning disorder, and ASD. M-CHAT, Modified Checklist of Autism in Toddlers; df: degree of freedom; AGFI, adjusted goodness-of-fit index; RMSEA, root mean square error of approximation.

SEM was used to investigate the pathway relationship of high-risk familial characteristics, children's developmental conditions, autism screening, and diagnosis of LD, DD, ADHD, and ASD. The model resulted in a good fit, with p = 0.424 (>0.05), AGFI 0.999 (>0.9), and RMSEA <0.001 (<0.08) (Figure 1). Pathway analysis showed that high-risk familial characteristics were associated with ADHD and ID, with those from high-risk families more likely to be diagnosed with ADHD and ID (β = 0.02, *p* < 0.001; β = 0.01, *p* = 0.036). Children with high-risk family characteristics were also more likely to be screened positive for ASD (β = 0.01, *p* = 0.042), and delayed developmental conditions at 1.5, 3, and 5.5 years (β = −0.07, *p* < 0.001; β = −0.05, *p* < 0.001; β = −0.02, *p* = 0.003). Girls were more likely to be screened as being at high risk of ASD (β = 0.02, *p* < 0.001); however, more boys were diagnosed with ADHD and ASD at 5.5 years (β = −0.04, *p* < 0.001; β = −0.04, *p* < 0.001). A higher risk of ASD and developmental conditions in children at ages 1.5, 3, and 5.5 were associated with a diagnosis of ADHD, ID, LD, and ASD. Children who scored >14 on M-CHAT were at higher risk of LD, ID, ADHD, and ASD (β = 0.15, *p* < 0.001; β = 0.08, *p* < 0.001; β = 0.02, *p* = 0.004; β = 0.08, *p* < 0.001). Delayed development in 1.5-year-old children increased their risk of a diagnosis of LD and ID (β = −0.06, *p* < 0.001; β = −0.11, *p* < 0.001). Delayed development at age 3 was related to an increased risk of being screened positive for ASD, and diagnosis of LD, ID, and ASD (β = −0.10, *p* < 0.001; β = −0.11, *p* < 0.001; β = −0.11, *p* < 0.001; β = −0.06, *p* < 0.001). Finally, delayed development at age 5.5 years increased the risk of being screened positive for ASD, and diagnosis of LD, ID, ADHD, and ASD (β = −0.29, *p* < 0.001; β = −0.24, *p* < 0.001; β = −0.25, *p* < 0.001; β = −0.11, *p* < 0.001; β = −0.09, *p* < 0.001).

## Discussion

In the national birth cohort dataset of 19,185 children, 2070 (10.8%) met at least one of the high-risk familial characteristics, including parents currently unmarried, parents unemployed, parents with no social insurance, parents who perceive a very heavy economic burden of childcare, or one or both parents have disability certification. Children who met any of the five high-risk familial characteristics were at increased risk for ADHD, ID, LD, and ASD. Children who met any of the five high-risk familial factors had a 1.21-fold increased risk for ADHD diagnosis, 1.36-fold increased risk for LD diagnosis, and 1.80-fold increased risk for ASD diagnosis, compared to children from families without familial risk factors. Increased relative risks were found for those who met two or more of the high-risk characteristics, showing a dose effect for high-risk characteristics. Pathway analysis showed that high-risk familial characteristics directly increased the risk for diagnosis of ADHD and ID, and indirectly increased the risk for diagnosis of ADHD, ID, LD, and ASD, through M-CHAT screening and children's developmental conditions. There was a gender disparity in that more girls were screened as being at risk of ASD though more boys were diagnosed with ADHD and ASD.

Pathway analysis showed that high-risk familial characteristics collected even as young as 1.5 years of age indicated an increased risk of a diagnosis of ADHD at 5.5 years of age. Overall family adversity (marital discord, low social class, large family size, paternal criminality, and maternal mental disorder), low family cohesion, and family relationships are associated with a higher risk of ADHD-inattentive type (Pheula et al., 2011). Family and parental dysfunction are strongly associated with psychopathy in children (Ingoldsby et al., 2001; D'Onofrio and Emery, 2019). Meta-analysis showed that post-divorce parental conflicts are associated with lower levels of support, structuring, and parent-child relationship quality, leading to more internalizing and externalizing problems, and lower levels of social adjustment and self-esteem in children (van Dijk et al., 2020), increasing their risk for developing psychopathology (Burt et al., 2008). Furthermore, studies on child protection court proceedings in the US and Canada have both found parents with a disability to be disproportionately involved in the child protection system (McConnell et al., 2010; Lightfoot and Slayter, 2014). Even with income control, parents with disabilities were 2.5 times more likely to engage in violence against their children, and this was due to their own childhood experiences of maltreatment, experiences of foster care, and witness to interpersonal violence (Minde et al., 2003). Therefore, parents with disability certification increase the risk of children being diagnosed with ADHD.

It is well established that ADHD is a familial and highly heritable disorder, with elevated rates in parents of children with ADHD, and the other way around (Minde et al., 2003). Adults with ADHD are more likely to encounter frequent job changes, marital breakdown, and higher rates of divorce (Wilens et al., 2004; Asherson et al., 2007). Parental ADHD is also associated with more severe clinical presentation of ADHD in their children, although whether this is due to environmental risk or gene-environment interaction is still unknown (Agha et al., 2013). Our longitudinal study found that high-risk familial characteristics expressed at age 1.5 increased the risk of a diagnosis of ADHD at 5.5 years both directly and indirectly; however, it is unknown whether the parents meet the diagnosis of ADHD.

Although no direct association was found between high-family risk characteristics and diagnosis of LD and ASD, children from high-risk families were more likely to have delayed development, which increased their risk of a diagnosis of LD and ASD. Therefore, children from high-risk families are indirectly at higher risk of diagnosis with ID, LD, and ASD. Parent and family dysfunction is associated with higher psychopathology in general (Wallander et al., 2006). Similar to the foregoing discussion of ADHD, it is hard to distinguish the cause and consequences of psychopathology, because genetic and environmental transactional processes can increase the risk of pediatric pathology, which in turn can increase the stress on family members and impair their functioning (Wallander et al., 2006). Systematic review and meta-analysis have shown parents of children with ASD and/or developmental delay perceived greater parenting stress than children from other clinical groups (Barroso et al., 2018). In the same line, a previous study also found mothers of children with respiratory diseases, compared to mothers of typically developing children, perceived greater levels of stress, lower self-esteem, more external locus of control, and poorer memory performance (Smirni et al., 2019). These studies support the possible transactional process of children's chronic illness and parenting stress. It has been found that parents of children with ASD suffered more psychopathologically and perceived less marital satisfaction and family adaptation and cohesion than mothers of typically developing children (Gau et al., 2012). Family dysfunction also increases the stress on mothers of children with ASD (Zaidman-Zait et al., 2017).

A dose effect in high-risk familial characteristics was found, with those meeting more risk characteristics being at an increased relative risk of diagnosis. Furthermore, children's developmental condition at 3 years was predictable for the diagnosis of ADHD, ID, LD, and ASD at 5.5 years old. Previous studies of TBCS-DI have shown development at 1.5 years and later to be predictive of children's emotional, cognitive, and social-communicative development at age eight (Lung et al., 2020). However, when the diagnosis is put into context, children's developmental trajectories show that language development is unstable before 3 years, and therefore not suitable for ASD diagnosis (Lung and Shu, 2021).

There was a gender disparity in that more girls were screened as being at risk of ASD, but more boys were diagnosed with ADHD and ASD in the pathway analysis. ADHD and ASD are both more commonly diagnosed in males (American Psychiatric Association, 2013). A 4.20:1 male-to-female prevalence for ASD was found in a meta-analysis (Loomes et al., 2017), and a similar gender ratio of 4:1 was found for the diagnosis of ADHD in the community (Ramtekkar et al., 2010). Other studies have shown that females are underdiagnosed, and even those who have been diagnosed with ADHD, where they display fewer externalized behavioral problems, are also less likely to be prescribed medication (Mowlem et al., 2019). Since the nature and etiology of ASD have been described as being the extreme male brain, diagnostic gender stereotype bias may be perpetuated by professionals, decreasing their sensitivity to autistic symptoms in girls (Bargiela et al., 2016). The results of our study support the idea that females may be underdiagnosed, and girls are more likely to be screened as being at risk of ASD but less likely to be diagnosed with ADHD or ASD.

A limitation of this study was that all information was parent-reported, including children's diagnoses. The constraint of using a national birth cohort dataset is that since a variety of information about children's development and health is collected, only limited information about each aspect can be obtained. The use of a psychometrically sound instrument for the assessment of familial risk factors or sensory processing and executive functions of children (such as the Assessment of Sensory Processing and Executive Functions in Childhood) may provide us with more specific information regarding how children's daily activities are affected by these diagnoses (Romero-Ayuso et al., 2018). Furthermore, more information regarding parents' pathology conditions may provide us with more information regarding the gene-environment interaction of these diagnoses. However, the strength of the national birth cohort datasets is that longitudinal follow-up information from each developmental stage is collected, decreasing the possibility of recall bias.

In summary, we used a large national birth cohort dataset, with familial risk characteristics collected at age 1.5, and followed children up to 5.5 years, to investigate whether high-risk families had an increased risk of a diagnosis of LD, ID, ADHD, and ASD. High-risk family characteristics (including parents currently unmarried, parents unemployed, parents with no social insurance, parents who perceive a very heavy economic childcare burden, and one or both parents have disability certification) increased the risk of LD, ID, and ADHD, with a dose effect of more high-risk characteristics leading to a higher risk of diagnosis. High-risk family characteristics directly increased the risk of a diagnosis of ADHD and ID, and indirectly increased the risk of a diagnosis of ADHD, ID, LD, and ASD, through the mediating factor of children's development. Furthermore, children's development at 3 years old was predictive of diagnosis at 5.5 years. Thus, developmental screening at age of three is important in early developmental interventions. Furthermore, the path to parenting, and preventive family-focused and/or child-rearing services for at-risk families are vital to improved outcomes for these children and their families.

## Data availability statement

Taiwan Birth Cohort Study datasets can be applied from the Taiwan Ministry of Health and Welfare, Bureau of Health Promotion. Requests to access the datasets should be directed to https://dep.mohw.gov.tw/DOS/np-2500-113.html.

## Ethics statement

The studies involving human participants were reviewed and approved by Ministry of Health and Welfare, Taipei, Taiwan. Written informed consent to participate in this study was provided by the participants' legal guardian/next of kin.

## Author contributions

L-JS and B-CS overlooked the sampling and experimental procedures. F-WL and P-FC undertook the statistical analysis and interpreted the analysis. P-FC wrote the first draft of the manuscript. All authors designed the study and contributed to and have approved the final manuscript.

## Funding

This study was supported by grants from the Health Promotion Administration, Ministry of Health and Welfare, Taiwan (DOH95-HP-1802, DOH96-HP-1702, and DOH99-HP-1702).

## Conflict of interest

The authors declare that the research was conducted in the absence of any commercial or financial relationships that could be construed as a potential conflict of interest.

## Publisher's note

All claims expressed in this article are solely those of the authors and do not necessarily represent those of their affiliated organizations, or those of the publisher, the editors and the reviewers. Any product that may be evaluated in this article, or claim that may be made by its manufacturer, is not guaranteed or endorsed by the publisher.
